# Quackery in France

**Published:** 1859-03

**Authors:** 


QUACKERY IN FRANCE.—THE PHYSICIANS DEMAND PROTECTION OF THE GOVERNMENT.—THEIR PETITION TO THE EMPEROR.—SIR—At an epoch in which the history of your benevolenee is written in ineffaceable characters on the face of France; at the moment in which society, imitating your example, is striving to ameliorate the condition of the poor classes, the medical corps deem it proper to call the attention of your Majesty to one of the social evils which paralyze your generous intentions.

Sir, along side of that medicine consecrated by the experience of ages; of that medicine which consoles and cures, there boldly marches an ignorant, sordid and illegal medicine.

If, in the cities, the instruction of the masses can sometimes counteract its grievous effects, it is not so in the country, where unfortunately ignorance still predominates, and where it lavishes its scandalous and deceitful promises with unrestrained publicity.

Sir, there is in this a serious danger, a barrier which constantly impedes that impulsion which you so freely give to morality and humanity.

Permit us to hope that your majesty, so careful of the children of the poor, and so provident for the aged poor, will deign to pursue this work by protecting the whole community by an efficacious repression against the illegal practice of medicine.

Such, sir, are the wishes of the medical corps, so often called to the honor of propagating the benefits of your touching solicitude for the suffering, but too often fettered by prejudice in the accomplishment of its mission of humanity.

We have the honor to be, with the most profound respect, the very humble and the very devoted servants of your majesty.

*Paris*, Sept. 1858. [*Signatures.*]

The above is a copy of the petition of the deputies of the medical societies of the Seine to the emperor, who received it with great complaisance, and even with gratification. The emperor expressed surprise that the practice of medicine was not more effectually protected by existing laws, and enquired if it were possible that remedies were advertised in the public journals, and sold to the public, without having first received the sanction of the Academy of medicine; and he promised that the petition should be sent to the proper minister with special instructions to examine it and give it the attention that its importance demanded.—*Nashville Med. Jour.—Ib.*

